# Transmission of antimicrobial resistance genes from the environment to human gut is more pronounced in colorectal cancer patients than in healthy subjects

**DOI:** 10.1002/imt2.70008

**Published:** 2025-03-05

**Authors:** Weixin Liu, Harry C. H. Lau, Xiao Ding, Xiaole Yin, William Ka Kei Wu, Sunny Hei Wong, Joseph J. Y. Sung, Tong Zhang, Jun Yu

**Affiliations:** ^1^ Institute of Digestive Disease and The Department of Medicine and Therapeutics State Key Laboratory of Digestive Disease, Li Ka Shing Institute of Health Sciences, CUHK Shenzhen Research Institute, The Chinese University of Hong Kong Hong Kong SAR China; ^2^ Environmental Microbiome Engineering and Biotechnology Laboratory, Center for Environmental Engineering Research, Department of Civil Engineering The University of Hong Kong Hong Kong SAR China; ^3^ Lee Kong Chian School of Medicine Nanyang Technological University Singapore Singapore

**Keywords:** antimicrobial resistance gene, colorectal cancer, environment, horizontal gene transfer

## Abstract

Antimicrobial resistance is a major global health concern. However, the source of gut resistome remains unsolved. We aimed to analyze the contribution of environmental antimicrobial resistance genes (ARGs) to colorectal cancer (CRC) patients. Here, we collected metagenomic data from 1,605 human stool samples (CRC = 748; healthy = 857) and 1,035 city‐matched environmental samples, in which 110 CRC, 112 healthy, and 56 environmental samples were newly collected. Compared to healthy subjects, CRC patients had significantly higher ARG burden (*p* < 0.01) with increased levels of multidrug‐resistant ARGs. Gut ARGs in CRC also had a closer similarity to environmental ARGs (*p* < 0.001). By comparing environmental and gut ARGs, 28 environmental ARGs were identified as CRC‐specific ARGs, including *SUL2* and *MEXE*, which were not identified in healthy subjects. Meanwhile, more mobile ARGs (mARGs) from the environment were observed in CRC patients compared to healthy subjects (*p* < 0.05). The hosts of mARGs were mainly pathogenic bacteria (e.g., *Escherichia coli* (*E. coli*) and *Clostridium symbiosum* (*C. symbiosum*)). Compared to healthy subjects, CRC patients showed elevated horizontal gene transfer efficiency from the environment to gut. Consistently, the abundance of pathobionts carrying specific mARGs (e.g., *E. coli‐SUL2* and *C. symbiosum‐SUL2*) were significantly increased in CRC patients compared to healthy subjects (*p* < 0.05). We thus reveal a route of ARG dissemination from the environment into the gut of CRC patients.

## Highlights

- Colorectal cancer (CRC) patients have a higher antimicrobial resistance gene (ARG) burden compared to healthy subjects.
- ARG profile between the city‐matched environment and human gut is more similar in CRC patients than in healthy subjects.
- CRC patients had more environment‐gut co‐shared ARGs, more mobile ARGs (mARGs), and higher ARG transmission efficiency from the environment to gut, compared to healthy subjects.
- Bacterial hosts of mARGs were mainly pathogenic bacteria (e.g., *Escherichia coli* and *Clostridium symbiosum*), which were significantly enriched in CRC patients compared to healthy subjects.

## Graphical abstract

Our study collected city‐matched environmental (*n* = 1,035) and human stool samples (colorectal cancer (CRC) = 748; healthy subjects = 857) worldwide to investigate the environmental resistome in CRC. Compared to healthy subjects, CRC patients had significantly higher antimicrobial resistance gene (ARG) burden with a higher similarity to environmental ARG profile. Additionally, CRC patients had more environment‐gut co‐shared ARGs, more mobile ARGs (mARGs), and higher ARG transmission efficiency from the environment to gut, compared to healthy subjects. The hosts of these mARGs were mainly pathogenic bacteria (e.g., *Escherichia coli* and *Clostridium symbiosum*). We thus revealed a route of mARG dissemination from the environment to human gut in CRC.

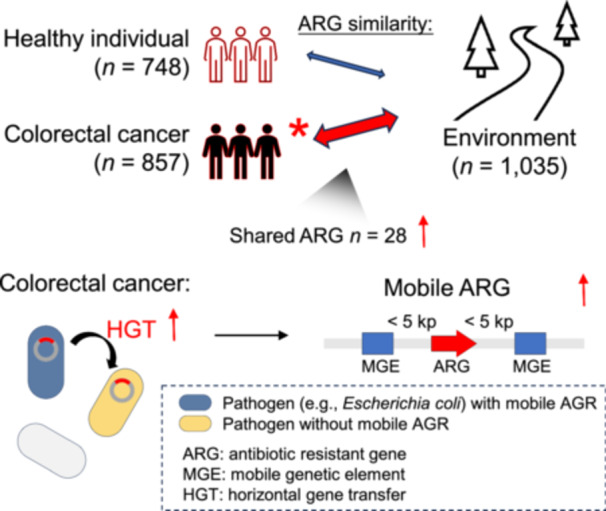

## INTRODUCTION

Antimicrobial resistance is one of the top global public health concerns [1, 2]. The widespread dissemination of antibiotic resistance genes (ARGs) in human pathogens has posed preeminent clinical challenges, especially in the management of infection [3, 4]. High antimicrobial resistance burden can cause failure in antibiotics to effectively eliminate bacteria, potentially leading to severe infection, worsened patient outcomes, and increased mortality [5, 6]. Colorectal cancer (CRC) is the second most deadly cancer worldwide [7], and gut pathobionts play a pivotal role in the initiation and progression of colorectal tumorigenesis [8]. However, it is largely unclear if CRC patients have a higher carriage of antibiotic resistance and whether the abundance of ARG‐carrying gut pathobionts is increased in CRC.

Humans and their living environment are closely connected and interdependent [1, 2]. The immense diversity of the environmental microbiome can serve as a gene reservoir for human pathogens to acquire resistance and counteract the effect of antibiotics [9]. It was reported that antimicrobial resistance is developed in humans as a consequence of exposure to polluted environments such as wastewater, contaminated food, and other sources that contain antimicrobial‐resistant bacteria [10]. More specifically, gut pathobionts and commensal bacteria can acquire ARGs via horizontal gene transfer (HGT) from environmental sources, leading to the development of antibiotic resistance [11]. Mechanistically, HGT relies on mobile genetic elements (MGEs), such as bacterial plasmids and phages, which can carry ARGs and facilitate their transfer from the environment to humans [12]. Hence, evaluating the risk of environment‐gut transmission of ARGs is important to gain insights into the connectivity between the environment and human CRC.

In this study, we collected 1,605 human stool samples (CRC = 748; healthy subjects = 857) and 1,035 city‐matched environmental samples with shotgun metagenomic sequencing data for analyses (in which 110 CRC, 112 healthy, and 56 environmental samples were newly collected). We found that CRC patients had significantly higher ARG burdens and a higher similarity to environmental ARG profiles than healthy subjects. More mobile ARGs (mARGs) from environment were observed in CRC patients compared to healthy subjects. The hosts of these mARGs were mainly pathogenic bacteria (e.g., *Escherichia coli* (*E. coli*), *Klebsiella pneumoniae* (*K. pneumoniae*), and *Clostridium symbiosum* (*C. symbiosum*)). Moreover, we discovered that the transmission efficiency of ARGs from the environment to human gut was increased in CRC, and the abundance of pathobionts carrying mobile ARGs was significantly enriched in CRC patients compared to healthy subjects.

## RESULTS

### CRC patients have a higher ARG burden compared to healthy subjects

**Figure 1:**
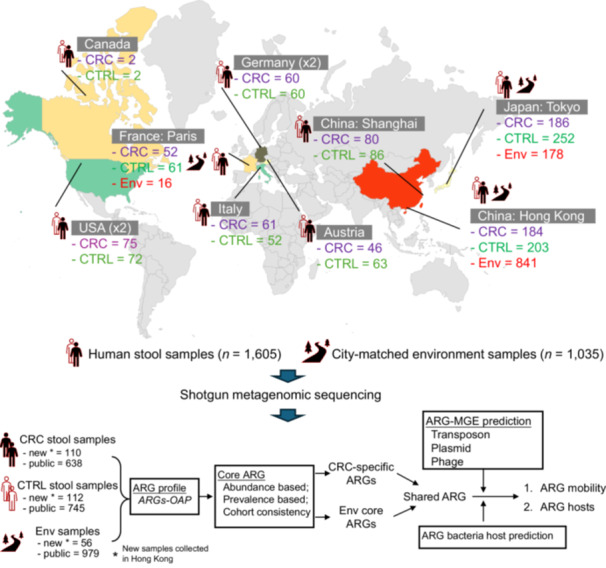
Study design and analysis workflow. (panel: up) Human stool samples from colorectal cancer (CRC) patients and healthy subjects, and city‐matched environment samples were collected worldwide. (panel: down) Summary of the workflow including analysis of core antimicrobial resistance gene (ARG) identification, ARG mobility, and ARG bacterial hosts. ARG profile was measured by *ARGs‐OAP* tool. CTRL, healthy controls; Env, environment; MGE, mobile genetic element. [Correction added on 11 March 2025, after first online publication: The Figure 1 of this paper has been updated at the author's request.]

**Figure 2:**
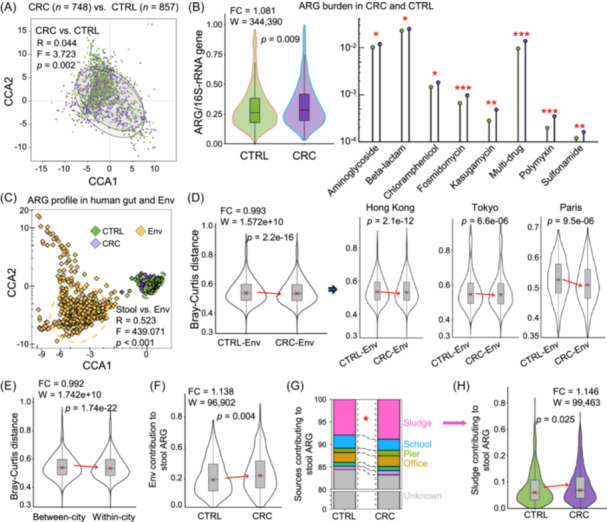
Convergence of the human gut and environmental antimicrobial resistance genes (ARGs) in colorectal cancer (CRC) patients. (A) Overall ARG profile between CRC patients and healthy subjects. Significance was tested by permutational multivariate ANOVA (PERMANOVA). (B) Overall ARG burden (normalized by 16S‐rRNA gene level) (panel left) and resistant‐specific ARG burden (panel right) between CRC patients and healthy subjects. Fold change (FC) in median was calculated. (C) ARG profiles between human gut and environmental samples. Significance was tested by PERMANOVA. (D) Intra‐city (i.e., Hong Kong, Tokyo, and Paris, in Figure 1) environment‐gut ARG dissimilarity (measured by Bray‐Curtis distance) in CRC patients was lesser than in healthy subjects (left panel: all three cities; right panel: each city). (E) In CRC, intra‐city environment‐gut ARG dissimilarity was significantly less than inter‐city environment‐gut ARG dissimilarity. (F) The city‐matched environmental ARGs had greater impacts on CRC patients than on healthy subjects. Source tracking is measured by fast expectation‐maximization microbial source tracking (*FEAST*) method. (G) The impacts of different city‐matched environmental sources (i.e., sludge of wastewater treatment, swabs from school, pier, office) on CRC patients and healthy subjects. (H) ARG from city‐matched sludge had a greater impact on CRC patients than on healthy subjects. Significance was tested by a two‐tailed Wilcoxon Rank Sum test. *p < 0.05; **p < 0.01; ***p < 0.001. CCA, constrained correspondence analysis; FC, fold change.

A total of 1,605 individuals (748 CRC patients and 857 healthy subjects) and 1,035 environmental samples with shotgun metagenomic sequencing data were analyzed (Figure 1 and Tables S1–3). We first compared the gut ARG profile between CRC patients and healthy subjects. Correspondence analysis revealed that the gut resistome (determined by overall ARG profile) in CRC patients was significantly different from healthy subjects (*F* = 3.723, *p *= 0.002, permutational multivariate ANOVA (PERMANOVA)) (Figure 2A, Figure S1A). Gut ARG burden, as measured by the total ARG level normalized by 16S‐rRNA gene level (Figure 2B) or bacterial cell count (Figure S1B), was significantly higher in CRC patients compared to healthy subjects (*p* = 0.009, two‐tailed Wilcoxon Rank Sum test). Increased ARG burden in CRC patients could be validated in nine out of ten individual cohorts (Figure S1C), thus confirming that CRC is associated with higher ARG burden.

Next, we assessed the risks associated with antibiotic consumption on the human gut resistome. No subjects with antibiotic exposure within 3 months were included in this study. The gut resistome in subjects with antibiotic exposure (>3 months ago) was not significantly altered compared to those without antibiotic exposure (Figure S1D–E), consistent with the previous publication [13]. Furthermore, our analysis of ARG similarity between environmental samples and human subjects revealed a convergence of the environmental and gut resistome in CRC patients, irrespective of antibiotic exposure (Figure S1F). These results suggest that the increase in ARG burden among CRC patients is at least partially unrelated to antibiotic use occurring more than 3 months prior.

### Gut ARG profile in CRC patients is more similar to the environmental ARG profile compared to healthy subjects

The potential link between environmental ARGs and human gut ARGs was examined. Correspondence analysis revealed a marked difference between the environmental ARG profile and gut ARG profile, regardless of disease conditions (*F* = 439.07, *p* < 0.001, PERMANOVA) (Figure 2C). In addition, the overall environmental ARGs varied significantly across different environmental samples (*F* = 33.06, *p* < 0.001, PERMANOVA) (Figure S1G–I). We then compared ARG profile in stool samples from CRC patients or healthy subjects to city‐matched environmental samples (i.e., Hong Kong, Tokyo, and Paris) (Figure 1). The difference between environmental ARGs and gut ARGs was significantly less in CRC compared to healthy subjects from the same city (*p* = 2.2e‐16) (Figure 2D, Figure S2A), indicating that ARG profile in CRC is more similar to the environmental ARG profile. Moreover, the difference in environment‐gut ARGs was significantly lower between CRC and city‐matched environmental samples (i.e., within city), in comparison between CRC and environmental samples from unmatched cities (i.e., between city) (*p* = 1.74e‐22) (Figure 2E, Figure S2B). A similar observation was found for the environment‐gut ARG similarity based on resistant‐specific gene profiles (e.g., multidrug‐resistant ARGs) (Figure S2C). We further utilized fast expectation‐maximization microbial source tracking (*FEAST*) to quantitate the contribution of environmental ARGs [14], and identified that environmental ARGs had greater impacts on gut ARGs in CRC patients than in healthy subjects (*p* = 0.004) (Figure 2F, Figure S3). Among all city‐matched environmental sources (sludge of wastewater treatment, flocked swabs from residential, office, school, pier, railway station), sludge samples were the only environmental source that significantly contributed to the elevation of gut ARGs in CRC patients, compared to healthy subjects (*p* = 0.025) (Figure 2G,H). Taken together, we provided evidence of the contribution of environmental resistome from sludge to the increased ARG burden in CRC.

### CRC‐specific ARGs are shared with core environmental ARGs

**Figure 3:**
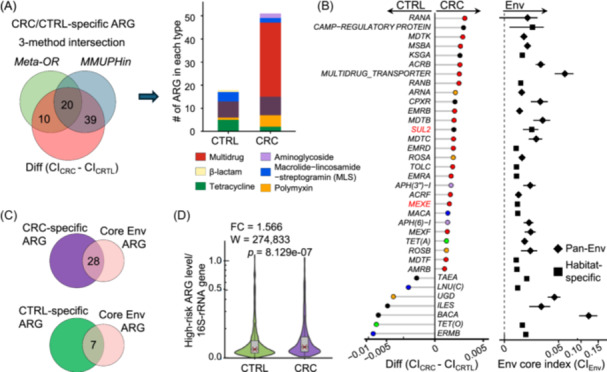
Colorectal cancer (CRC)‐specific antimicrobial resistance genes (ARGs) are shared with core environmental ARGs. (A) The differential ARGs between CRC patients and healthy subjects were detected by three methods (prevalence‐based meta‐analysis based on odd ratio (*Meta‐OR*), abundance‐based *MMUPHin*, and core index (CI)). Shown in the right panel was the distribution of resistant‐specific ARGs. (B) CRC‐/healthy‐specific ARGs (panel left; node color represents the type of ARG) were shared with core environmental ARGs (panel right; node shape represents whether the ARG is pan‐Env core ARG or habitat‐specific core ARG). The difference in core index was calculated. (C) The amount of shared ARGs between CRC‐/healthy‐specific ARGs and core environment ARGs. (D) A higher level of high‐risk ARGs (environment‐gut co‐shared) was found in CRC patients than in healthy subjects. ARG level was normalized by 16S‐rRNA gene level. Fold change (FC) in median was calculated. Significance was tested by a two‐tailed Wilcoxon Rank Sum test. Diff (CI_CRC_ − CI_CTRL_): difference in core index.

To identify differential ARGs between CRC patients and healthy subjects, we used two established methods (meta‐analysis based on odd ratio (*Meta‐OR*) (Table S4) and *MMUPHin* (Table S5)) and developed a core index (Figure S4A–C). By overlapping ARGs identified from these methods, a total of 69 ARGs were differentially distributed between CRC and healthy subjects (Figure 3A, Figure S4D). Among them, 51 ARGs were significantly enriched in CRC patients (Figure 3A), including *MEXE* (associated with hospitalization [4]) and *SUL2* (associated with sulfonamide drug resistance) (Figure 3B). The same core index was utilized to identify core environmental ARGs (Figure S5A, B). A total of 140 core environmental ARGs were identified (Figure S5C), and 57 of them were specifically present in sludge, while the remaining 83 were found in diverse environmental sources (Table S6).

By comparing environmental ARGs to human gut ARGs, we found that 28 core environmental ARGs were CRC‐specific ARGs (e.g., *MEXE* and *SUL2*), and 7 were healthy‐specific ARGs (e.g., *BACA*) (Figure 3B,C). To assess the health risk of ARGs, we employed an omics‐based framework [15] to classify ARGs into four categories (Rank I‐IV) risk framework (Figure S5D). We found that 25 out of 28 environment‐CRC co‐shared ARGs were present in this risk framework, and these high‐risk ARGs were significantly higher in CRC than in healthy subjects (Figure 3D, Figure S5E). Hence, the presence of environment‐CRC co‐shared ARGs further confirmed the similarity between the environmental and gut ARG profiles in CRC patients.

### CRC patients have more mobile ARGs than healthy subjects

**Figure 4:**
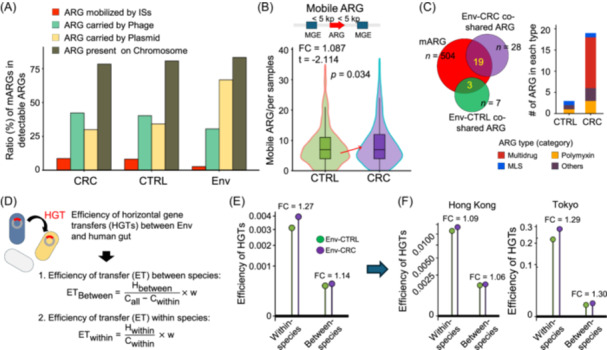
Mobile ARG (mARGs) in the human gut and environment. (A) The ratio of ARGs that (1) mobilized by ISs elements, (2) carried by plasmid, or (3) phage, and 4) present on bacterial chromosome in detectable ARGs of colorectal cancer (CRC) gut, healthy gut, and environment, respectively. (B) More mARGs were detected in CRC patients than in healthy subjects. Fold change (FC) in mean was calculated. Significance was tested by a two‐tailed Student's *t*‐test. (C) More environment‐gut co‐shared mARGs were identified in CRC patients than in healthy subjects. (D) Method for calculating mARG transmission efficiency within the same species (within‐species) or across different bacteria (between‐species). (E) Overall environment‐gut transmission efficiency of within‐species and between‐species ARGs. (F) City‐matched (i.e., Hong Kong and Tokyo, respectively) environmental‐gut ARG transmission efficiency was higher in CRC patients than in healthy subjects. The efficiency fold change (FC) between the CRC patients and healthy individuals was calculated. ET, efficiency of HGT; HGT, horizontal gene transfer; ISs, insertion sequences.

Given the coexistence of environmental and gut ARGs in CRC, we hypothesized that part of the gut resistome was derived from the environment by HGT. We therefore developed a pipeline incorporating ARG profile and different databases to predict ARG‐associated MGEs, including insertion sequences (ISs), bacterial plasmid, and virus/phage (Figure S6A). We observed that more environmental ARGs were carried by bacterial plasmids than phages, in contrast to gut ARGs, which were more likely to be carried by phages (Figure 4A). Compared to healthy subjects, CRC patients had more mobile ARGs (*p* = 0.034) *(*Figure 4B, Figure S6B), and 19 environment‐CRC co‐shared ARGs were identified as mobile ARGs including *MEXE* and *SUL2* (Figure 4C and Table S7).

### ARG transmission efficiency from the environment to gut is higher in CRC patients than in healthy subjects

Transmission of ARGs (i.e., HGT) can occur not only within the same species but also across less related microbes [16]. We therefore evaluated the transmission efficiency of ARGs from the environment to human gut within the same species or across different bacteria (Figure 4D). As expected, the transmission efficiency was markedly higher within the same species compared to the efficiency among different species (Figure 4E). On the other hand, the transmission efficiency of both within‐species and between‐species ARGs was higher from the environment to CRC than to healthy gut (Figure 4E), regardless of environmental source (Figure S6C and Table S8). The higher environment‐gut ARG transmission efficiency in CRC than in healthy gut can be validated in both Hong Kong and Tokyo (two cities with sufficient sample size) (Figure 4F). Collectively, our findings showed that the increased ARG burden in CRC is at least in part owing to high ARG transmission efficiency from the environment to the gut of CRC patients.

### The hosts of mobile ARGs are opportunistic pathogenic bacteria

Microbes (especially pathogens) serve as a reservoir of ARGs and are key determinants of disseminating antibiotic resistance. To this end, we determined the bacterial host of ARGs in environment and human gut by metagenomic assembling (Figure S6A). A total of 140,372 metagenome‐assembled genomes (MAGs) were constructed, and 77,214 of them were species‐level MAGs that belong to 794 different species (Tables S9, 10). Species‐level MAGs were then classified into three categories based on their quality: high‐quality (*n* = 8,086; completeness ≥ 90, contamination < 5, strain heterogeneity < 0.5), median‐quality (*n* = 6,565; 50 ≤ completeness < 90, contamination < 5), and the remaining low‐quality (*n* = 62,473) (Figure S7A, B and Table S10). We found that genomes with high and median quality were comparable to reference genomes from the National Center for Biotechnology Information (NCBI) genome database (Figure S7C). Hence, these high/median‐quality genomes were used to determine the amount of mARGs they carried.

**Figure 5:**
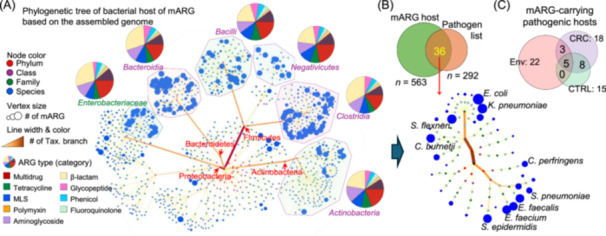
Bacterial hosts of mobile ARGs (mARGs). (A) Phylogenetic tree of bacterial hosts of mARG. The vertex represents the bacterial host, and its size represents the normalized number of mARG it carried. Specific clades including *Bacteroidia*, *Bacilli*, *Negativicutes*, *Clostridia*, *Actinobacteria*, and *Enterobacteriaceae*, were circled. The pie charts represent the distribution of resistant‐specific mARGs. (B) Number of mARG‐carrying pathobionts. Panel down: phylogenetic tree of pathobionts including *Escherichia coli* (*E. coli*) and *Klebsiella pneumoniae* (*K. pneumoniae*). (C) More mARG‐carrying pathobionts (environment‐gut co‐shared) were identified in CRC patients than in healthy subjects. CRC, colorectal cancer.

A total of 563 bacterial hosts of mARGs were identified, which were mainly *Clostridia*, *Bacilli*, *Bacteroidetes*, and *Gammaproteobacteria* (especially the *Enterobacteriaceae* family) (Figure 5A). The majority of mARGs carried by these bacteria belonged to β‐lactam, multidrug, and aminoglycoside‐resistant ARG subtypes (Figure 5A, Figure S7D). Based on the Pathogen Host Interactions Database (PHI‐base) [17] and the list of antibiotic‐resistant “priority pathogens” from the World Health Organization (WHO), 36 bacterial hosts of mARG with pathogenic potential were identified, including *E. coli* and *K. pneumoniae* (Figure 5B). In particular, more pathogenic hosts of mARG were found in CRC patients than in healthy subjects (Figure 5C). In addition, among all environmental sources, sludge samples shared the highest number of pathogenic mARG bacterial hosts with the gut in CRC (Figure S7E), implying the positive correlation between wastewater and CRC.

### mARG‐carrying pathobionts are enriched in CRC patients compared to healthy subjects

**Figure 6:**
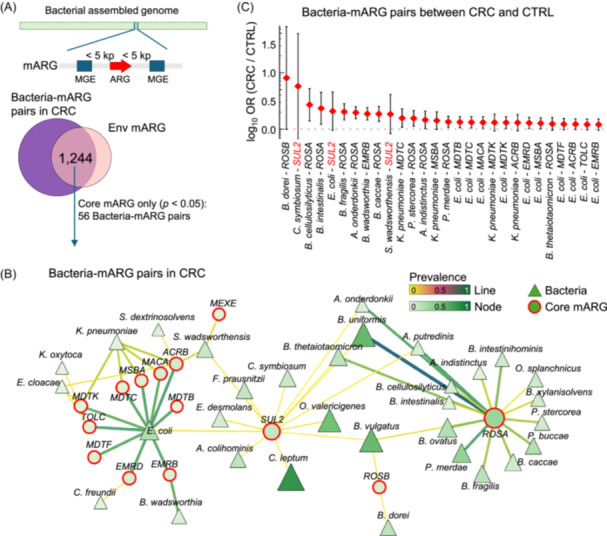
Mobile ARG (mARG)‐carrying pathobionts (bacteria‐mARG pair) are enriched in colorectal cancer (CRC) patients compared to healthy subjects. (A) Panel up: bacteria hosts and mARGs (environment‐gut co‐shared) are paired up using our pipeline (Figure S6A) (bacteria‐mARG pair); Panel down: 1,244 bacteria‐mARG pairs were detected in CRC. (B) The network represents significant bacteria‐mARG pairs (*n* = 56) consisting of pathobionts and environment‐CRC co‐shared mARGs (Figure 4C). (C) 28 bacteria‐mARG pairs are enriched in CRC patients compared to healthy subjects. Odd ratio (OR) between the CRC and CTRL was calculated. Significance was tested by a Fisher exact test. *p* < 0.05 were considered as statistically significant. OR, odd ratio.

We next paired up bacterial hosts and mARGs (i.e., bacteria‐mARG pairs), and compared these mARG‐carrying pathobionts between CRC patients and healthy subjects. In the CRC gut, we identified a total of 1,244 bacteria‐mARG pairs in which mARGs co‐existed in the environment (Figure 6A, Figure S8A). Among them, 56 significant bacteria‐mARG pairs consisting of pathobionts (identified based on the Pathogen Host Interactions database and the list of “priority pathogens” from WHO) and environment‐CRC co‐shared mARGs (Figure 4C), for example *E. coli* carrying the mARG *SUL2* (*E. coli‐SUL2*), were identified (Figure 6B). Compared to healthy subjects, 28 bacteria‐mARG pairs such as *E. coli‐SUL2*, *C. symbiosum‐SUL2*, and *Sutterella wadsworthensis‐SUL2* were significantly enriched in CRC patients (*p* < 0.05, Fisher exact test) (Figure 6C and Table S11). The abundances of several pathogenic bacterial hosts of mARGs, including *E. coli* and *K. pneumoniae*, were not significantly changed in CRC patients (Figure S8B), implying the elevated level of some mARGs in CRC patients (Figure 3B) might be unrelated to bacterial expansion. Taken together, our results revealed that mARG‐carrying pathobionts could be transmitted from the environment, causing their enrichment in the gut of CRC patients.

## DISCUSSION

The role of gut microbiome in CRC is well‐established, but the impact of antimicrobial resistance acquired from the environment on this malignancy remains undetermined. Here, to our understanding, this is the first meta‐analysis that deciphers the association of environmental ARGs resistome with CRC. Our multi‐cohort analysis reported a higher ARG burden in CRC patients than in healthy subjects. Human health is closely connected to microbes in the surrounding environment. Microbes can acquire ARGs from polluted environments (e.g., soil, water). Transmission of these ARG‐carrying microbes facilitates the development of antimicrobial resistance in humans, thereby reducing the efficacy of antibiotics against infection and increasing the burden of hospital care [6]. Our analysis showed a closer similarity between environmental and gut ARG profiles in CRC patients than in healthy subjects, and this was validated by the source‐tracking algorithm. Moreover, we identified 28 core environmental ARGs that were significantly enriched in CRC, in comparison to seven environment‐healthy co‐shared ARGs. The mobility of ARGs was also elevated in CRC patients compared to healthy subjects. Our in‐silico analysis, therefore, illustrated that environmental ARGs could be horizontally transferred into the human gut to reshape the gut ARG profile in CRC patients.

We revealed that ARGs in human gut are partially derived from the environment through HGT. In general, HGT heavily relies on the presence of MGEs such as ISs, bacterial plasmids, and phages. MGEs usually encode traits to adapt to the changing environment and serve as carriers to traffic foreign DNA between bacterial cells [12]. Meanwhile, ARG can be carried by MGEs, thus enabling its transmission from the environment to humans. Through metagenomic assembling, we identified a diverse distribution of ARG‐carrying MGEs in the environment, particularly showing that more environmental ARGs were carried by plasmids than phages. Certain human activities, such as extensive use of antibiotics in the livestock industry, aquaculture, and clinical settings, exert selection pressure on environmental bacteria [18]. This pressure compels these bacteria to acquire new functions through MGEs to rapidly adapt to changing conditions. Research suggests that plasmids often carry ARGs to enhance the survival of their bacterial hosts, which in turn positively contributes to host fitness [19, 20]. Conversely, phages can impose significant costs on their hosts, causing 10%–20% of daily bacterial mortality and over 200% during bacterial expansion [21]. Additionally, lysogenic phages can be reactivated under certain conditions, such as antibiotic treatment [22]. Hence, this dynamic helps explain the higher prevalence of plasmids carrying ARGs in the environment.

ARGs that were mobilized by MGEs in the environment and human gut were then evaluated. We identified 19 environment‐CRC co‐shared mARGs, which is greater than the number of environment‐healthy co‐shared mARGs (*n* = 3). Consistently, the transmission efficiency of ARGs between the environment and humans was higher in CRC patients than in healthy subjects, regardless of environmental source (Figure S6C). Healthy individuals and patients with CRC from the same city are exposed to similar environmental risks. However, healthy individuals with a robust gut microbiome and normal immune function exhibit greater resistance to colonization of ARG‐carrying bacteria and other pathogens. In contrast, cancer patients are more susceptible to infection due to their immunocompromised state and diminished capacity to combat opportunistic pathogens [23]. Furthermore, patients with cancer face a higher likelihood of acquiring bacterial infections during frequent hospital visits and stays, which increases their risk of hospital‐acquired infections. Our analysis revealed significant similarity in ARGs between sludge samples and the gut microbiome of CRC patients. This may be explained by the very close distance between the hospital for human sample collection and the river for sludge sample collection in our in‐house cohort. Together, these findings suggest that exposure to contaminated environments, such as wastewater and hospital waste, may facilitate the transfer of ARGs to humans, potentially elevating ARG levels in CRC patients.

Our analysis showed that more pathogenic bacteria are shared between the environment and CRC patients compared to healthy subjects (Figure 4G), including *E. coli* that produces several genotoxins to break host double‐stranded DNA in CRC [8], and *C. symbiosum* that activates hedgehog signaling in CRC through producing branched‐chain amino acids [24]. mARGs carried by *E. coli* or *C. symbiosum* (e.g., *SUL2*, associated with resistance to sulfonamide drug which is involved in diverse biological activities including anticancer [25]) were also significantly enriched in CRC patients. On the other hand, HGT can be driven by inflammation. It is well‐established that colorectal tumorigenesis is fueled by chronic intestinal inflammation [26]. Inflamed environments create favorable conditions for the bloom of oxygen‐tolerant and pathogenic species that are particularly prone to engage in HGT, such as *Enterobacteriaceae* identified in previous [27] and our studies (Figure 5A). Collectively, we identified ARG‐carrying pathobionts that might underline the increased transmission efficiency of ARGs from the environment into the gut of CRC patients.

This study has several limitations. First, our analysis is based on multiple retrospective cohorts; therefore, the major findings are comparative and could not establish causality between environmental ARGs and CRC tumorigenesis. Second, there are only three cohorts with city‐matched environmental data and human CRC samples, which limits the generalizability of our results regarding environmental ARGs in CRC. To enhance future investigations, it is essential to collect additional city‐matched environmental and human samples. Third, the functional roles of identified mARG‐carrying pathobionts and their impacts on CRC tumorigenesis remain undetermined in this study.

## CONCLUSION

In summary, we illustrated a route of mARG dissemination from the environment to human gut in CRC. Elevated transmission efficiency of ARGs leads to the enrichment of environmental mARGs in the gut, resulting in increased ARG burden in CRC patients. Altogether, our work provides new insights into the association of environmental resistome with CRC patients.

## METHODS

### Human stool samples

A total of 1,605 individuals comprising 748 CRC patients and 857 healthy subjects from our in‐house cohort (*n* = 220) and published fecal metagenomic datasets from 9 different countries/cities (*n* = 1,382) were included in this study (Figure 1 and Tables S1, 2). Fecal samples were collected from an in‐house CRC cohort (*n* = 220) consisting of 110 CRC patients and 112 healthy subjects, who received colonoscopy screening from Jockey Club Bowel Cancer Education Center, The Chinese University of Hong Kong. The exclusion criteria were: (1) use of antibiotics within the past 3 months; (2) on a vegetarian diet; (3) had an invasive medical intervention within the past 3 months; (4) had a past history of any cancer, or inflammatory disease of the intestine. No significant trend of antibiotic use in CRC patients preceding diagnosis was found compared to healthy subjects (Odd ratio = 0.93, *p* > 0.05; Fisher exact test). All subjects had intact colonic mucosa at the time of stool collection and signed informed consent form. The clinical study protocol was approved (No. CRE‐2010.198 and CRE‐2011.297) by the Joint Chinese University of Hong Kong–New Territories East Cluster Clinical Research Ethics Committee.

Additionally, we searched for CRC studies published from January 2014 to January 2023 on PubMed and found nine studies with shotgun metagenomic sequencing on human fecal samples. There were 1,382 human subjects consisting of 638 CRC patients and 745 healthy subjects. Sample size and details of sample collection are shown in Tables S1, 2. Metagenomic datasets were downloaded from European Nucleotide Archive (ENA) and DNA Data Bank of Japan using the following identifier: ERP005534 for Zeller et al. [28], ERP008729 for Feng et al. [29], PRJEB10878 for Yu et al. [30], PRJEB12449 for Vogtmann et al. [31], PRJNA389927 for Hanningan et al. [32], PRJEB27928 for Wirbel et al. [33], SRP136711 for Thomas et al. [34], PRJNA731589 for Liu et al. [35], and DRA006684 and DRA008156 for Yachida et al. [36].

### Environmental samples

We also collected 1,035 city‐matched environmental samples comprising sludge of wastewater treatment from our in‐house cohort (*n* = 56), and flocked swabs from residential (*n* = 344), office (*n* = 32), school (*n* = 110), pier (*n* = 174), and railway station (*n* = 172) retrieved from the MetaSUB Consortium [37] (Tables S1, 3). Sludge samples were collected from the hybrid moving bed biofilm reactor (HMBBR; a type of wastewater treatment process) in Hong Kong in 2021.

Additionally, to improve the representativeness of the environmental data, we collected more public available environmental samples including air (*n* = 81), soil (*n* = 198), water (*n* = 64), wastewater treatment plants (WWTP, *n* = 303), landfill (*n* = 26), and sediment (*n* = 261) from 65 countries/regions worldwide (Table S3 and Figure S1G).

### Shotgun metagenomic sequencing

Total DNA was extracted from fecal samples and sludge samples by QIAamp DNA Stool Mini Kit (Qiagen) and DNeasy PowerSoil Kit (Qiagen) according to the manufacturer's instructions, respectively. Shotgun metagenomic sequencing was performed at Novogene by Illumina sequencing platform with paired‐end 150‐bp.

### Profiling of antimicrobial resistance genes from bacteria

*KneadData* (v0.7.2) with default parameters was applied for quality control of all metagenomic sequencing data. This tool can eliminate contaminated reads of the host or other sources and retain only the microbial reads. *ARGs‐OAP* (v3.2.2) was used for ARG profiling with default settings [38]. The identified ARGs were then classified at resistant‐specific types or subtype levels and quantified by a universal unit by normalizing the read counts against the estimated cell counts or 16S rRNA gene copies. The cell counts or 16S rRNA gene copies (i.e., coefficients) were estimated either by mapping against an essential single‐copy marker gene database or by correcting for the copy numbers of 16S rRNA sequences. The ARG burden was defined as the sum of the normalized abundance of all identified ARGs. Bacterial taxonomic profiles were generated by *MetaPhlAn3* (v3.0.6) with default settings.

### Analysis for tracking the ARG sources in human fecal samples

*FEAST* [14] with default settings was used to estimate the impact of environmental sources on human fecal ARG profile and was conducted with the R package *FEAST* (v0.1.0).

### Core ARGs identification

We aimed to identify the core ARGs in the environment and human gut. To achieve this, we developed a core index (CI) considering the ARG prevalence, ARG abundance and ARG consistency among different cohorts or environmental sources. The proposed CI of the *i‐*th ARG was calculated as follows:

$${\text{CI}}_{\text{i}}\;=\;{\text{w}}_{\text{i}}\;\times \;{\text{RA}}_{\text{i}}\;\times \;{\text{F}}_{\text{i}}$$

CI difference (CI_diff_) for the *i‐*th ARG between CRC patients and healthy subjects was defined as:

$${\text{CI}}_{\text{diff}}\;=\;{\text{CI}}_{\text{CRC}}-{\text{CI}}_{\text{CTRL}},$$

where RA_*i*_ and *F_i_* are the relative abundance and prevalence of the *i‐*th ARG, respectively. *w_i_* is a factor considering the existence of *i‐*th ARG in different cohorts or environmental sources. ARG with prevalence > 25% in an individual cohort or environment source was considered as present on that cohort or environment source.

To see the significance of CI and CI_diff_, we obtained the overall CI density and CI_diff_ density, and fitted appropriate mathematical distributions on the densities according to the observed density shape, respectively. A *p* value was calculated by the cumulative probability p(CI≥q). The significance level α was set to 0.05.

### Metagenomic assembling and prediction of MGEs

We developed a pipeline to reconstruct the ARG‐associated MGEs. The metagenomic sequencing reads were first assembled with *MEGAHIT* (v1.2.8), to generate the bacterial genomic contigs; contigs with length <1 kb were excluded from further analysis. We then predicted the ARGs on contigs by using *DeepARG* (v1.0.1) and *RGI* (v5.1.0). The tool *DeepARG* is more accurate than other tools and can reduce the false negatives significantly [39]; whilst the tool *RGI* can annotate single nucleotide polymorphisms on the ARGs [40]. ARG databases including *CARD*, *ARDB*, and *UNIPROT* were used for the ARG annotation.

To predict MGEs, we first applied the *BLAST* (v2.9.0+) tool to annotate ISs or transposons on ARG‐carrying contigs using the *ISfinder* database. ARGs can also be carried by plasmids or phages, which facilitate ARG dissemination via conjugation, transposition, or transformation [12]. Therefore, we also predicted the presence of plasmids or phages in the ARG‐carrying contigs. *PlasFlow* (v1.1) was utilized for the plasmid prediction; whilst *VirSorter* (v1.0.6), *DeepVirFinder* (v1.0), and *geNomad* (v1.7.1) were utilized for the phage prediction. We incorporated all the genomic information, including the predicted ARGs, genomic location of ISs elements, and the contigs that were annotated as plasmids or phages, together to obtain the mobility of ARGs.

### Prediction of ARG bacterial hosts

Contigs were binned using *MaxBin* (v2.0) to obtain the drafted genomes. The genome quality of each bin was assessed via *CheckM* (v1.1.2) with lineage_wf presetting workflow using default parameters. Bins were labeled by quality according to the guidelines [41]: genomes with high‐quality (HQ) for those having completeness ≥ 90, contamination < 5, and strain heterogeneity < 0.5; genomes with median‐quality (MQ) for those having completeness < 90, completeness ≥ 50, and contamination < 5; and the remaining low quality (LQ) genomes. Furthermore, we retrieved the genomes of bacterial isolates from the NCBI genome database. Comparable results were found between the assembled genomes (with HQ and MQ) and genome references. The reconstructed genomes enable us to identify the presence of plasmids and phages in the bacterial genomes. The phylogenetic tree was constructed based on bacterial taxonomic information.

### Analysis of ARG transmission efficiency

We calculated the ARG HGT efficiency between the environment and human gut using a previous method [42] with minor modifications adjusting for the sample size. The ARG HGT efficiency was calculated based on data from the assembled bacterial genomes and mobile ARGs carried by these assemblies. Specifically, efficiency of transfer (ET) among bacterial cells of the same species was calculated by

$${\text{ET}}_{\text{within}}=\;\frac{{\text{H}}_{\text{within}}}{{\text{C}}_{\text{within}}}\times \text{w}.$$

The efficiency of transfer among bacterial cells of different species was calculated by

$${\text{ET}}_{\text{between}}\;=\;\frac{{\text{H}}_{\text{between}}}{{\text{C}}_{\text{All}}-{\text{C}}_{\text{within}}}\;\times \;\text{w},$$

where ${\text{H}}_{\text{within}}$ and ${\text{H}}_{\text{between}}$ represent the number of within‐species and between‐species environment‐gut HGT events, respectively. w is factor considering the sample size and was calculated by $\frac{1}{{\text{h}}_{1}\times {\text{h}}_{2}}$, where ${\text{h}}_{1}$ and ${\text{h}}_{2}$ represent the number of samples in habitats 1 and 2, respectively. ${\text{C}}_{\text{All}}$ and ${\text{C}}_{\text{within}}$ were calculated as

$${\text{C}}_{\text{All}}=\;\text{e}\;\times \;\text{g},$$

$${\text{C}}_{\text{within}}\;=\;\sum_{\text{i}}^{\text{n}}{\text{e}}_{\text{i}}\;\times \;{\text{g}}_{\text{i}}$$

where *e* and *g* are the number of genomes identified in the environment and human gut, respectively. ${\text{e}}_{\text{i}}$ and $g_{i}$ represent the number of genomes for *i‐*th species in the environment and human gut, respectively. The *n* in *C*_within_ represents the number of shared species between the environment and human gut.

### Statistical analysis

Data was presented as mean ± standard deviation (SD) or median (first quartile, third quartile) when appropriate. Data was tested by two‐tailed Student's *t*‐test or two‐tailed Mann–Whitney *U* test when appropriate. All analyses were conducted under the open‐source framework of R software (version 3.5.2). Meta‐analysis based on ARG abundance was carried out by *MMUPHin* (v1.16.0) method. Meta‐analysis based on ARG prevalence was carried out using *metafor* (v4.4‐0) package. Fisher exact test was used to evaluate the enrichment of mARG‐carrying bacteria in CRC patients compared to healthy subjects. All differences were considered statistically significant if *p* < 0.05. To account for multiple‐testing, *p* values were adjusted using Benjamini–Hochberg false discovery rate (FDR) correction.

## AUTHOR CONTRIBUTIONS

**Weixin Liu**: Investigation; data curation; formal analysis; visualization; writing—original draft. **Harry C. H. Lau**: Writing—review and editing. **Xiao Ding**: Data curation. **Xiaole Yin**: Resources. **William Ka Kei Wu**: Resources. **Sunny Hei Wong**: Resources. **Joseph J. Y. Sung**: Resources; Writing—review and editing. **Tong Zhang**: Writing—review and editing; resources; supervision. **Jun Yu**: Writing—review and editing; project administration; resources; conceptualization; supervision.

## CONFLICT OF INTEREST STATEMENT

The authors declare no conflicts of interest.

## ETHICS STATEMENT

The clinical study protocol was approved by Joint Chinese University of Hong Kong–New Territories East Cluster Clinical Research Ethics Committee (Nos. CRE‐2010.198 and CRE‐2011.297). All subjects had intact colonic mucosa at the time of stool collection and signed informed consent form.

## Supporting information


**Figure S1.** Overall ARG profile in the environment and human gut, as well as the effects of the subject's clinical information on the human gut ARG profile.
**Figure S2.** Intra‐city and between‐city environment‐gut ARG dissimilarity.
**Figure S3.** The impacts of environmental ARGs on human gut resistome.
**Figure S4.** ARG core index (CI) and differential CI in human samples.
**Figure S5.** ARG core index (CI) in environmental samples.
**Figure S6.** Analysis of ARG mobility in human samples.
**Figure S7.** The statistics of assembled genomes.
**Figure S8.** The bacteria‐mARG pairs in CRC and differential bacteria.

**Table S1.** Samples collected from worldwide (See Table S2 for details).
**Table S2.** Samples metadata for human stool.
**Table S3.** Samples metadata for environment (Env).
**Table S4.** Prevalence based differential analysis.
**Table S5.** Abundance based differential analysis (by *MMUPHin*).
**Table S6.** Pan‐Env and Env‐specific core ARGs.
**Table S7.** Core ARG (CRC‐specific ARGs co‐shared with Env ARGs) identified as mobile ARG (MGE ISs present in the up‐/down‐stream of the ARG sequence).
**Table S8.** ARG transmission efficiency from different environments to gut.
**Table S9.** Statistics of genome assembly, species detection with different assembly quality, and the number of ARG detected.
**Table S10.** Statistics of assembled species.
**Table S11.** CRC‐enriched Host ‐ARG pair and its associated MGE (ISs, phage or plasmid).

## Data Availability

The human stool sequencing data has been deposited in the Genome Sequence Archive (GSA) at https://ngdc.cncb.ac.cn/gsa/, with accession number CRA022416 (https://ngdc.cncb.ac.cn/gsa/search?searchTerm=CRA022416). The environmental sequencing data has been deposited in the GSA, with accession number CRA022431 (https://ngdc.cncb.ac.cn/gsa/search?searchTerm=CRA022431). The data and scripts used are saved in GitHub https://github.com/WilsonYangLiu/Environmental-ARGs-in-CRC-resistome.git. Supplementary materials (figures, tables, graphical abstract, slides, videos, Chinese translated version, and update materials) may be found in the online DOI or iMeta Science http://www.imeta.science/.
