# Supplementary material for: Transmission of antimicrobial resistance genes from the environment to human gut is more pronounced in colorectal cancer patients than in healthy subjects
Source: Imeta. 2025 Mar 5;4(2):e70008. doi: 10.1002/imt2.70008 (PMC11995172; doi:10.1002/imt2.70008)
Supplement: Supplementary file 1 — Figure S1. Overall ARG profile in the environment and human gut, as well as the effects of the subject's clinical information on the human gut ARG profile. Figure S2. Intra‐city and between‐city environment‐gut ARG dissimilarity. Figure S3. The impacts of environmental ARGs on human gut resistome. Figure S4. ARG core index (CI) and differential CI in human samples. Figure S5. ARG core index (CI) in environmental samples. Figure S6. Analysis of ARG mobility in human samples. Figure S7. The statistics of assembled genomes. Figure S8. The bacteria‐mARG pairs in CRC and differential bacteria. [file IMT2-4-e70008-s002.docx]

# Supporting information to

# Transmission of antimicrobial resistance genes from environment to human gut is more pronounced in colorectal cancer patients than healthy subjects

**Running title:** Environmental ARGs in CRC resistome

Weixin Liu^1^, Harry CH Lau^1^, Xiao Ding^1^, Xiaole Yin^2^, William Ka Kei Wu^1^, Sunny Hei Wong^3^, Joseph JY Sung^3^, Tong Zhang^2*^, Jun Yu^1*^

^1^Institute of Digestive Disease and The Department of Medicine and Therapeutics, State Key Laboratory of Digestive Disease, Li Ka Shing Institute of Health Sciences, CUHK Shenzhen Research Institute, The Chinese University of Hong Kong, Hong Kong SAR, China

^2^Environmental Microbiome Engineering and Biotechnology Laboratory, Center for Environmental Engineering Research, Department of Civil Engineering, The University of Hong Kong, Hong Kong SAR, China

^3^Lee Kong Chian School of Medicine, Nanyang Technological University, Singapore

*Correspondence: [junyu@cuhk.edu.hk](mailto:junyu@cuhk.edu.hk) (Jun Yu); [zhangt@hku.hk](mailto:zhangt@hku.hk) (Tong Zhang)


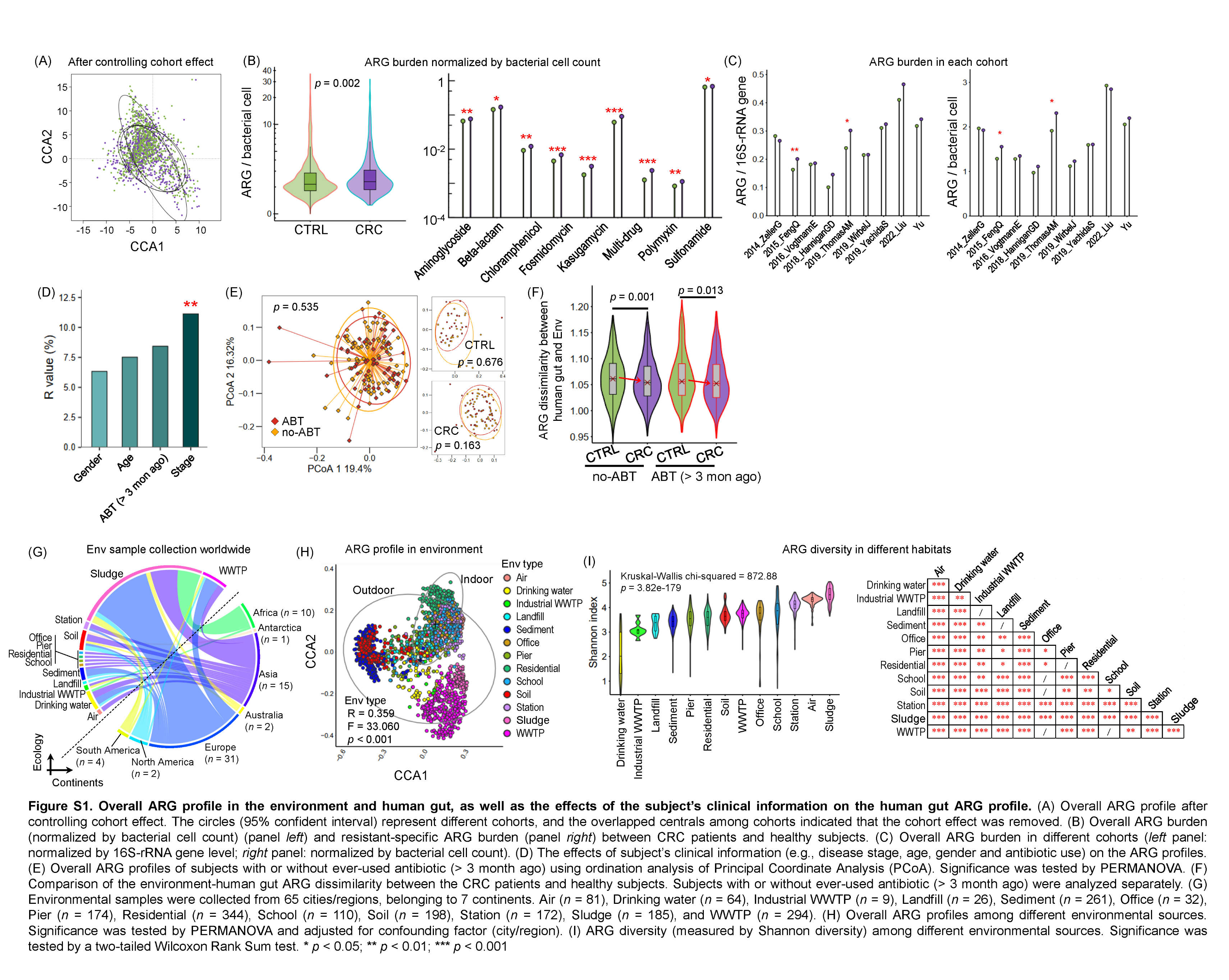


**Figure S1 Overall antibiotic resistance gene (ARG) profile in the environment (Env) and human gut, as well as the effects of the subject’s clinical information on the human gut ARG profile.** (A) Overall ARG profile after controlling cohort effect. The circles (95% confident interval) represent different cohorts, and the overlapped centrals among cohorts indicated that the cohort effect was removed. (B) Overall ARG burden (normalized by bacterial cell count) (panel left) and resistant-specific ARG burden (panel right) between colorectal cancer (CRC) patients and healthy subjects (CTRL). (C) Overall ARG burden in different cohorts (left panel: normalized by 16S-rRNA gene level; right panel: normalized by bacterial cell count). (D) The effects of subject’s clinical information (e.g., disease stage, age, gender and antibiotic use) on the ARG profiles. (E) Overall ARG profiles of subjects with or without ever-used antibiotic (> 3 month ago) using ordination analysis of Principal Coordinate Analysis (PCoA). Significance was tested by permutational multivariate ANOVA (PERMANOVA). (F) Comparison of the environment-human gut ARG dissimilarity between the CRC patients and healthy subjects. Subjects with or without ever-used antibiotic (> 3 month ago) were analyzed separately. (G) Environmental samples were collected from 65 cities/regions, belonging to 7 continents: Air (*n* = 81), Drinking water (*n* = 64), Industrial WWTP (*n* = 9), Landfill (*n* = 26), Sediment (*n* = 261), Office (*n* = 32), Pier (*n* = 174), Residential (*n* = 344), School (*n* = 110), Soil (*n* = 198), Station (*n* = 172), Sludge (*n* = 185), and WWTP (*n* = 294). (H) Overall ARG profiles among different environmental sources. Significance was tested by PERMANOVA and adjusted for confounding factor (city/region). (I) ARG diversity (measured by Shannon diversity) among different environmental sources. Significance was tested by a two-tailed Wilcoxon Rank Sum test. * *p* < 0.05; ** *p* < 0.01; *** *p* < 0.001. ABT: antibiotic; ARG: antimicrobial resistance gene; CCA: Constrained correspondence analysis; PCoA: principal coordinate analysis CRC: colorectal cancer; CTRL: healthy controls; Env: environment


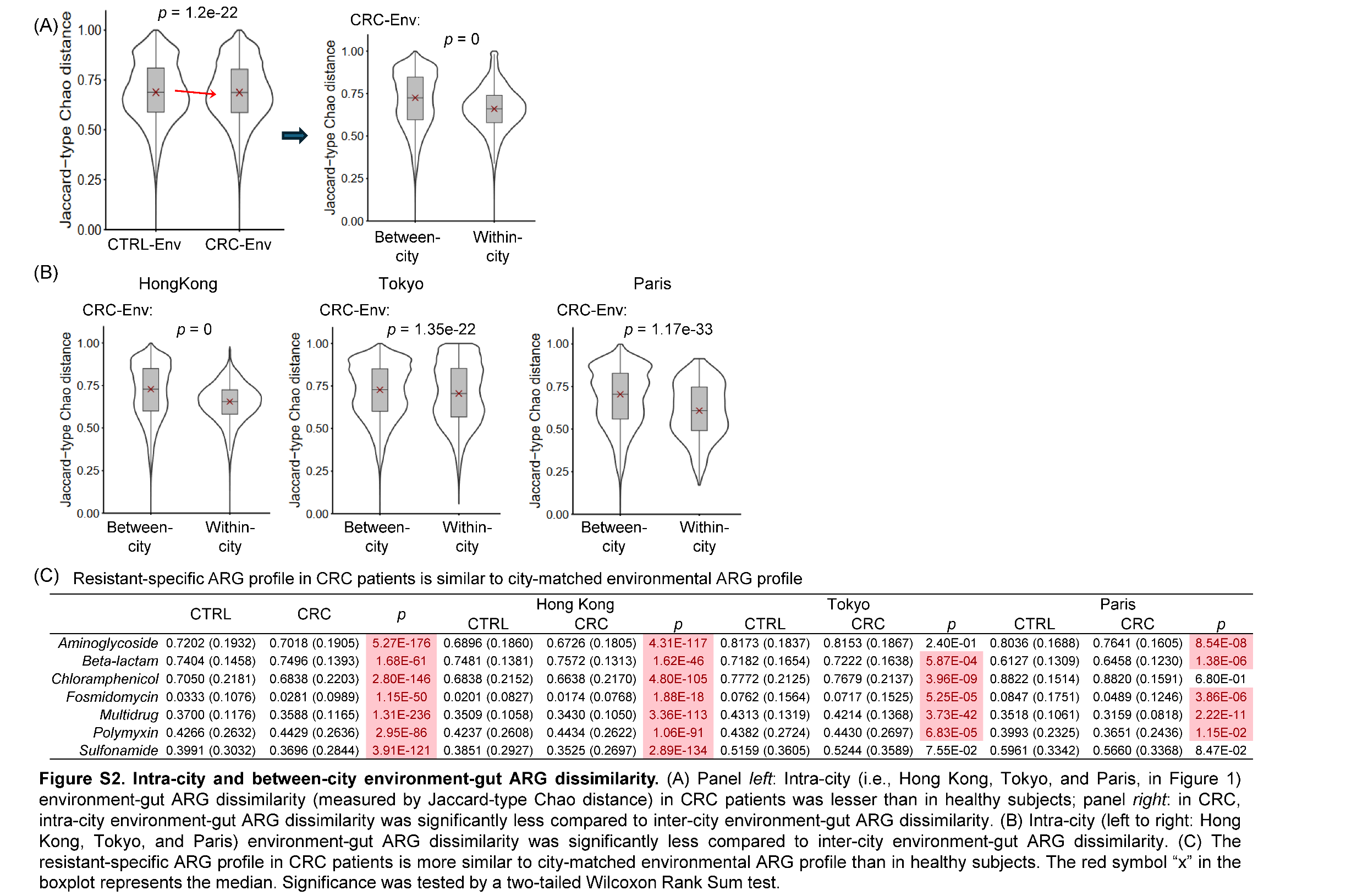


**Figure S2 Intra-city and between-city environment-gut ARG dissimilarity.** (A) Panel left: Intra-city (i.e., Hong Kong, Tokyo, and Paris, in Figure 1) environment-gut ARG dissimilarity (measured by Jaccard-type Chao distance) in CRC patients was lesser than in healthy subjects; panel right: in CRC, intra-city environment-gut ARG dissimilarity was significantly less compared to inter-city environment-gut ARG dissimilarity. (B) Intra-city (left to right: Hong Kong, Tokyo, and Paris) environment-gut ARG dissimilarity was significantly less compared to inter-city environment-gut ARG dissimilarity. (C) The resistant-specific ARG profile in CRC patients is more similar to city-matched environmental ARG profile than in healthy subjects. The red symbol “x” in the boxplot represents the median. Significance was tested by a two-tailed Wilcoxon Rank Sum test. ARG: antimicrobial resistance gene; CRC: colorectal cancer; CTRL: healthy controls; Env: environment.


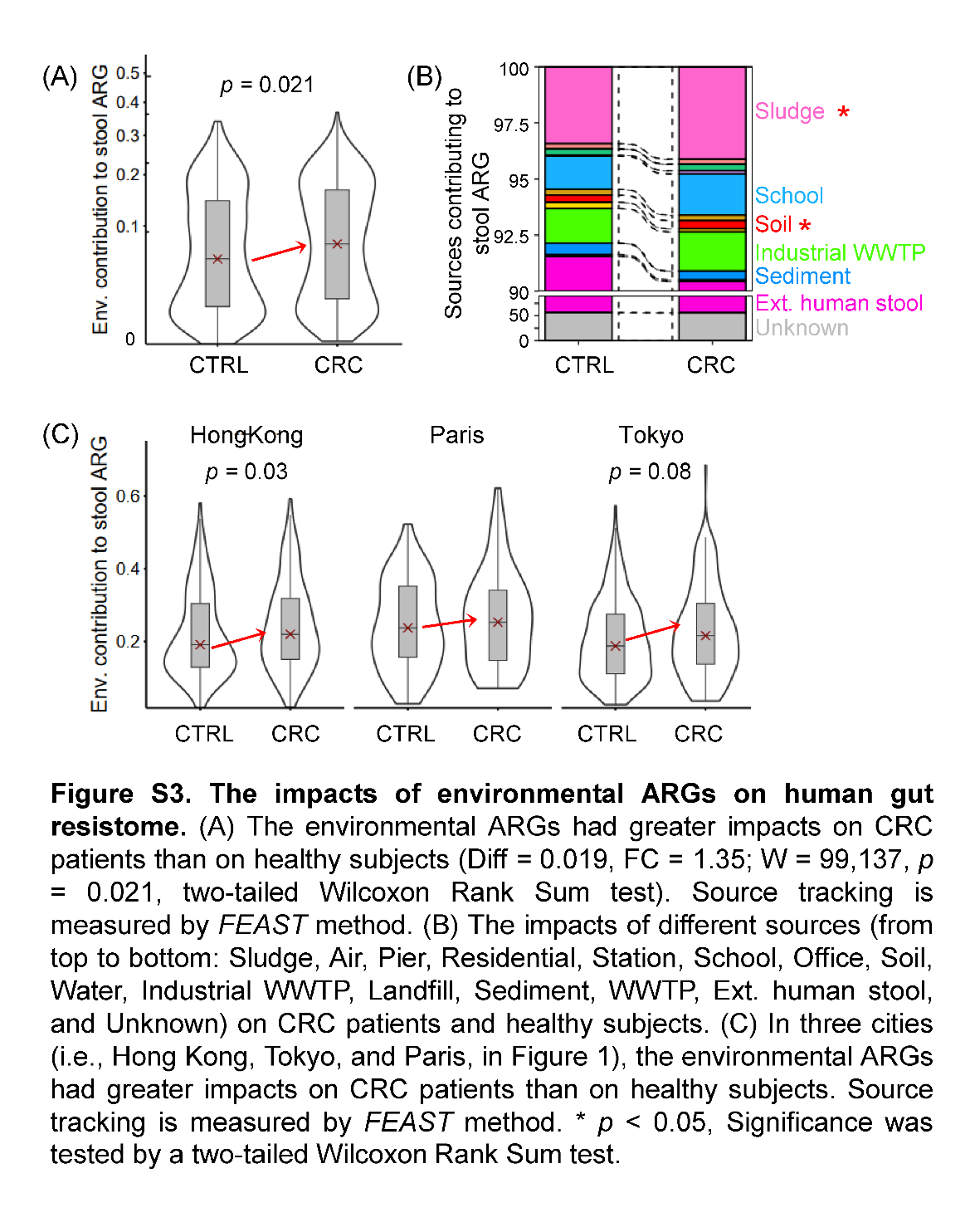


**Figure S3 The impacts of environmental ARGs on human gut resistome.** (A) The environmental ARGs had greater impacts on CRC patients than on healthy subjects (difference in median (Diff) *=* 0.019, FC = 1.35; W = 99,137, *p* = 0.021, two-tailed Wilcoxon Rank Sum test). Source tracking is measured by fast expectation-maximization microbial source tracking (*FEAST*) method. (B) The impacts of different sources (from top to bottom: Sludge, Air, Pier, Residential, Station, School, Office, Soil, Water, Industrial WWTP, Landfill, Sediment, WWTP, External (Ext.) human stool, and Unknown) on CRC patients and healthy subjects. (C) In three cities (i.e., Hong Kong, Tokyo, and Paris, in Figure 1), the environmental ARGs had greater impacts on CRC patients than on healthy subjects. Source tracking is measured by *FEAST* method. * *p* < 0.05, Significance was tested by a two-tailed Wilcoxon Rank Sum test. ARG: antimicrobial resistance gene; CRC: colorectal cancer; CTRL: healthy controls; Env: environment; Ext: external


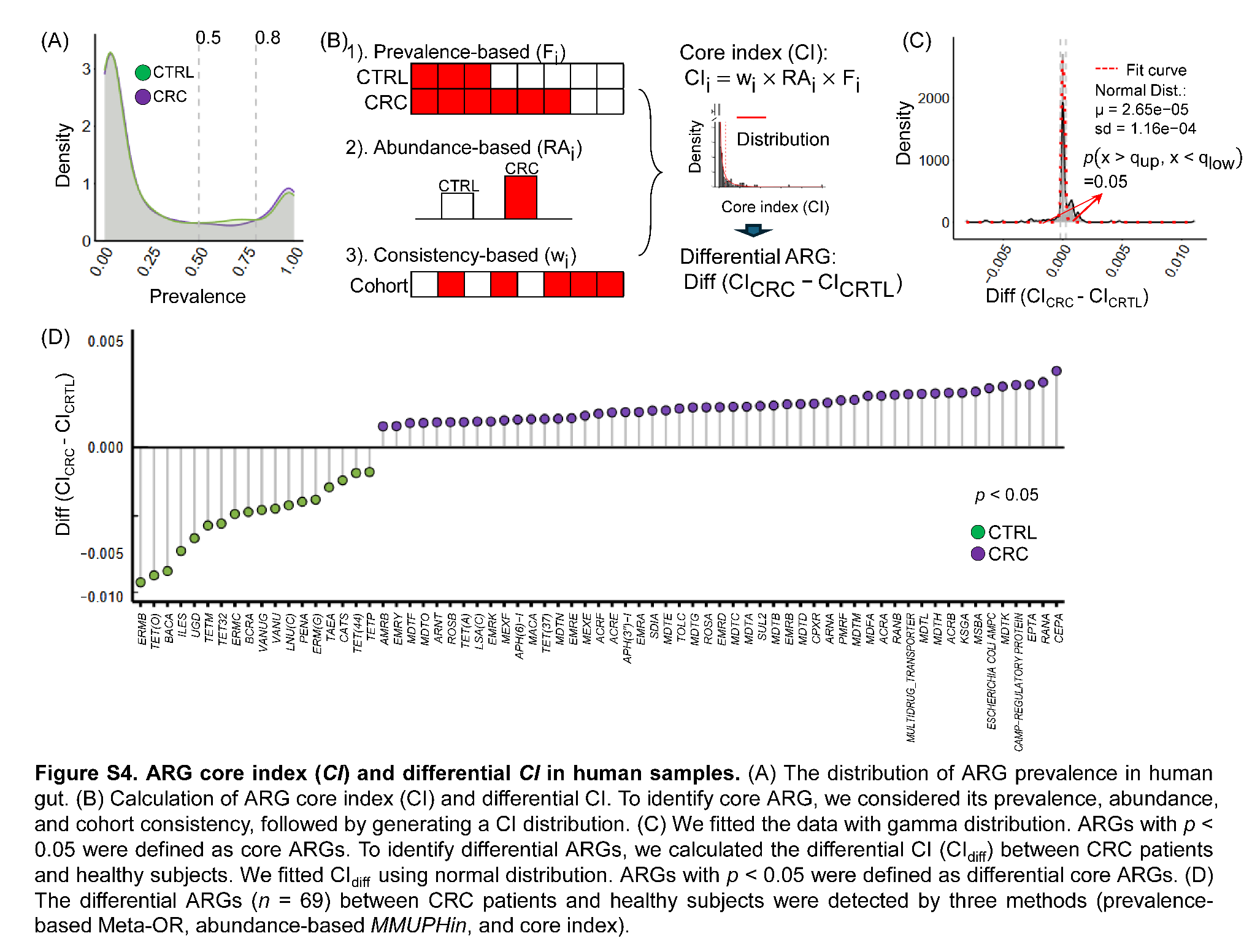


**Figure S4 ARG core index (CI) and differential CI in human samples.** (A) The distribution of ARG prevalence in human gut. (B) Calculation of ARG core index (CI) and differential CI. To identify core ARG, we considered its prevalence, abundance, and cohort consistency, followed by generating a CI distribution. (C) We fitted the data with gamma distribution. ARGs with *p* < 0.05 were defined as core ARGs. To identify differential ARGs, we calculated the differential CI (CIdiff) between CRC patients and healthy subjects. We fitted CIdiff using normal distribution. ARGs with *p* < 0.05 were defined as differential core ARGs. (D) The differential ARGs (*n* = 69) between CRC patients and healthy subjects were detected by three methods (prevalence-based meta-analysis based on odd ratio (*Meta-OR*), abundance-based *MMUPHin*, and core index). ARG: antimicrobial resistance gene; CI: core index; CRC: colorectal cancer; CTRL: healthy controls; Env: environment; Diff (CI_CRC_ - CI_CTRL_): difference in core index.


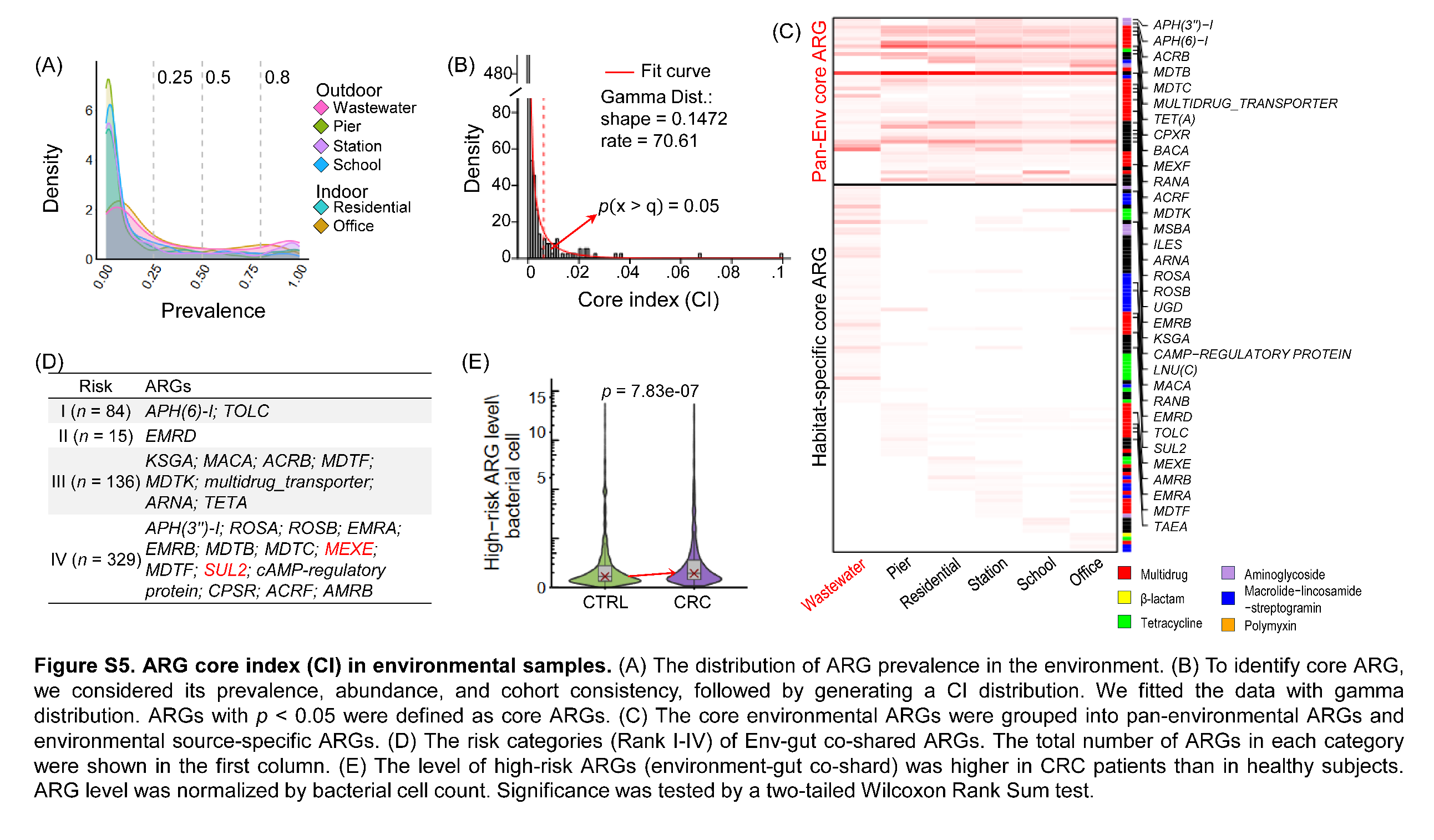


**Figure S5 ARG core index (CI) in environmental samples.** (A) The distribution of ARG prevalence in the environment. (B) To identify core ARG, we considered its prevalence, abundance, and cohort consistency, followed by generating a CI distribution. We fitted the data with gamma distribution. ARGs with *p* < 0.05 were defined as core ARGs. (C) The core environmental ARGs were grouped into pan-environmental (pan-Env) ARGs and environmental source-specific ARGs. (D) The risk categories (Rank I-IV) of Env-gut co-shared ARGs. The total number of ARGs in each category were shown in the first column. (E) The level of high-risk ARGs (environment-gut co-shard) was higher in CRC patients than in healthy subjects. ARG level was normalized by bacterial cell count. Significance was tested by a two-tailed Wilcoxon Rank Sum test. ARG: antimicrobial resistance gene; CI: core index; CRC: colorectal cancer; CTRL: healthy controls; Env: environment.


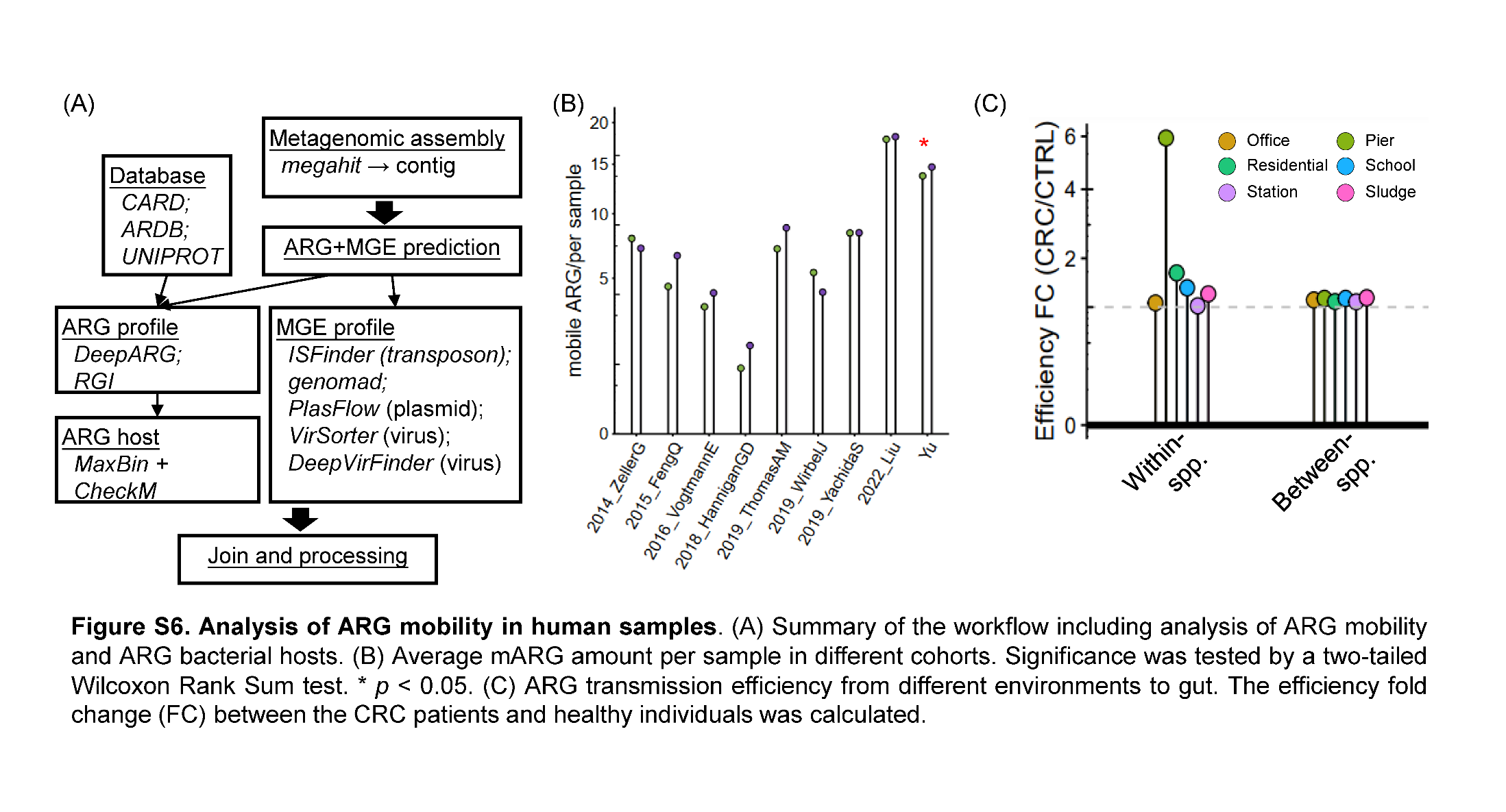


**Figure S6 Analysis of ARG mobility in human samples**. (A) Summary of the workflow including analysis of ARG mobility and ARG bacterial hosts. (B) Average mARG amount per sample in different cohorts. Significance was tested by a two-tailed Wilcoxon Rank Sum test. * *p* < 0.05. (C) ARG transmission efficiency from different environments to gut. The efficiency fold change (FC) between the CRC patients and healthy individuals was calculated. ARG: antimicrobial resistance gene; CRC: colorectal cancer; CTRL: healthy controls; Env: environment. MGE: mobile genetic element; FC: fold change; Within-/Between-spp.: within-/between species.


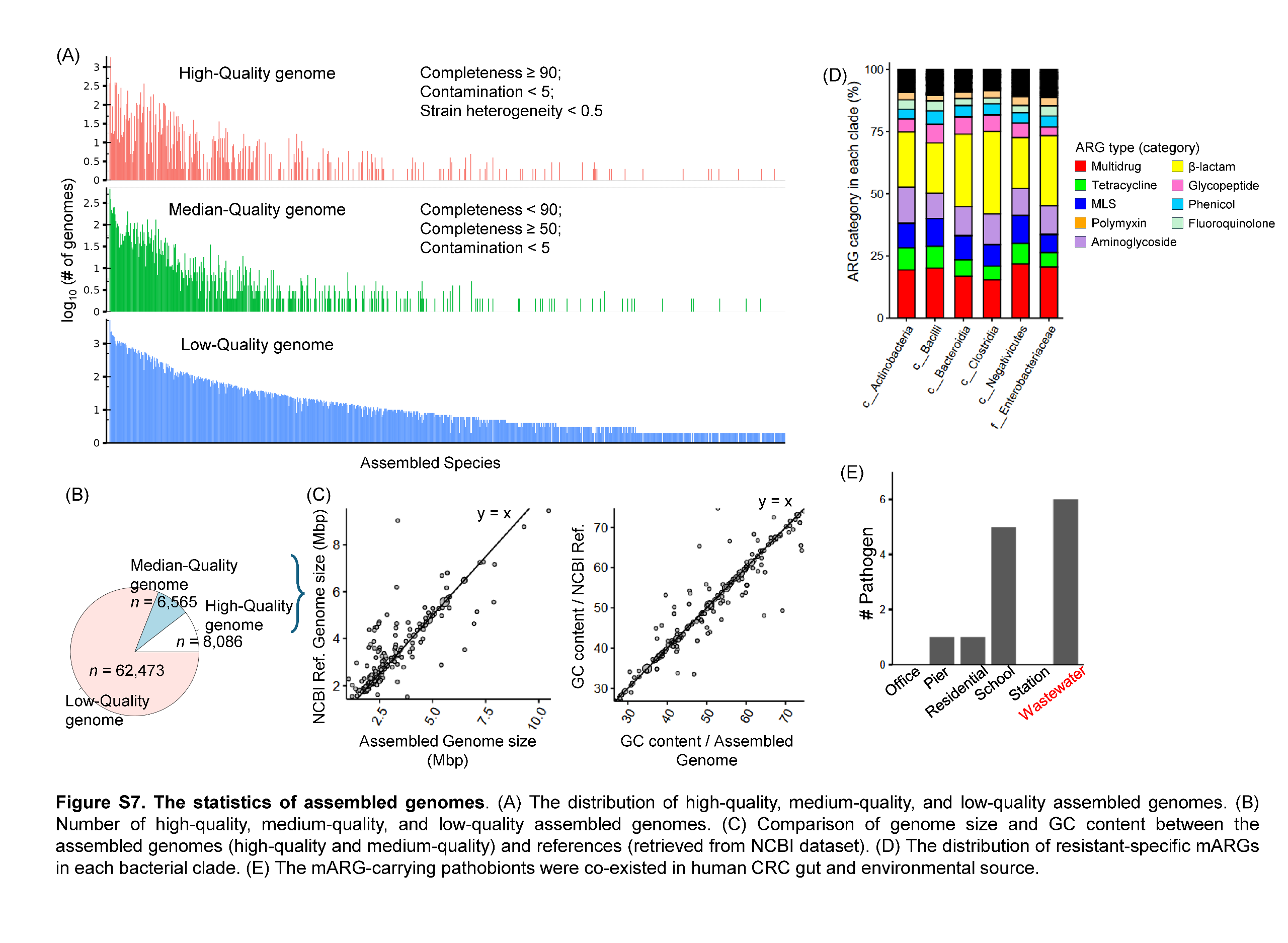


**Figure S7 The statistics of assembled genomes**. (A) The distribution of high-quality, medium-quality, and low-quality assembled genomes. (B) Number of high-quality, medium-quality, and low-quality assembled genomes. (C) Comparison of genome size and guanine-cytosine (GC) content between the assembled genomes (high-quality and medium-quality) and references (retrieved from NCBI dataset). (D) The distribution of resistant-specific mARGs in each bacterial clade. (E) The mARG-carrying pathobionts were co-existed in human CRC gut and environmental source. ARG: antimicrobial resistance gene; CRC: colorectal cancer; CTRL: healthy controls; GC: guanine-cytosine content; Ref: reference.


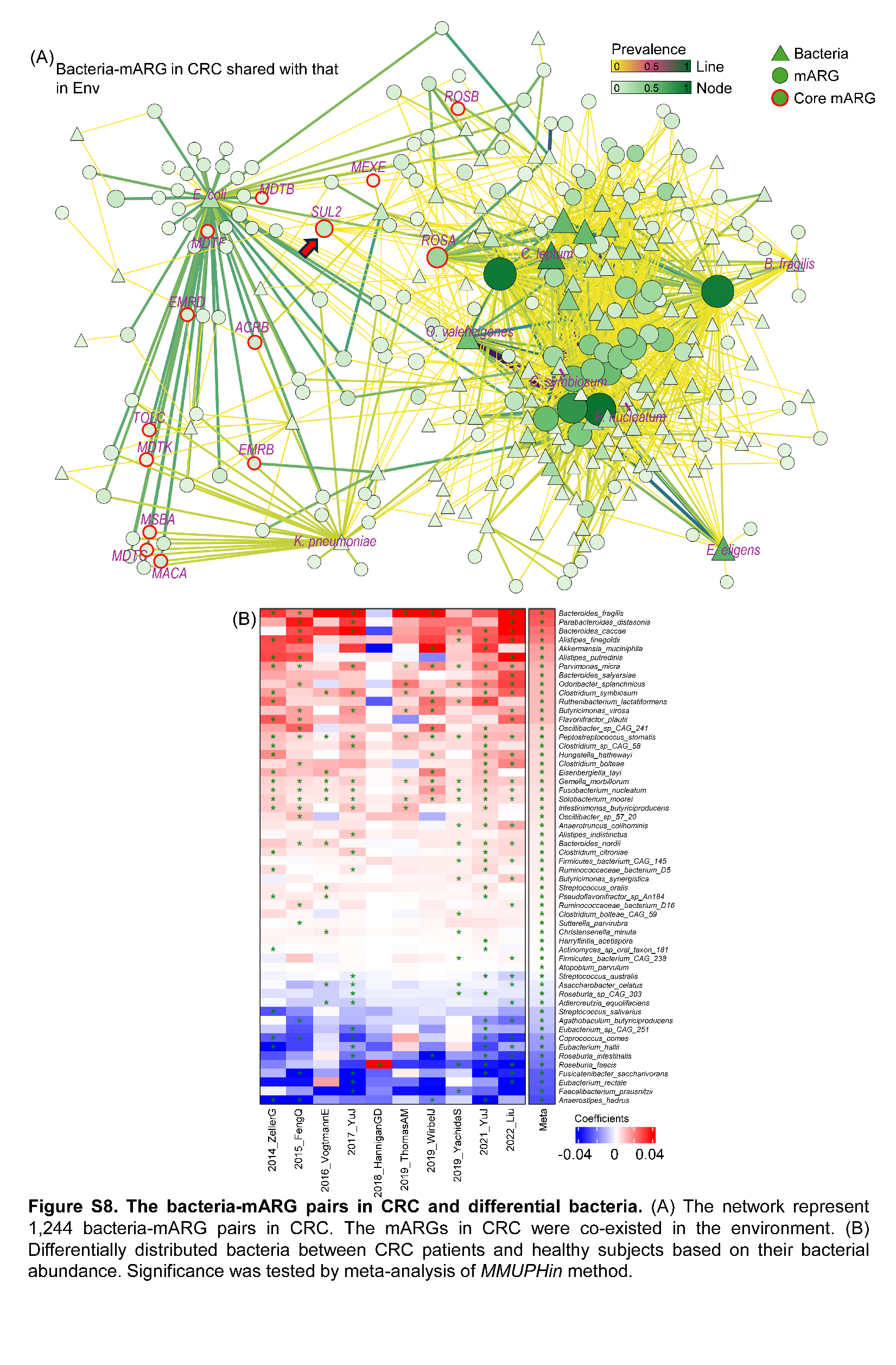


**Figure S8 The bacteria-mARG pairs in CRC and differential bacteria.** (A) The network represent 1,244 bacteria-mARG pairs in CRC. The mARGs in CRC were co-existed in the environment. (B) Differentially distributed bacteria between CRC patients and healthy subjects based on their bacterial abundance. Significance was tested by meta-analysis of *MMUPHin* method. ARG: antimicrobial resistance gene; CRC: colorectal cancer; CTRL: healthy controls. mARG: mobile ARG
